# Can individual fatty acids be used as functional biomarkers of dairy fat consumption in relation to cardiometabolic health? A narrative review

**DOI:** 10.1017/S0007114522000289

**Published:** 2022-12-28

**Authors:** Laury Sellem, Kim G. Jackson, Laura Paper, Ian D. Givens, Julie A. Lovegrove

**Affiliations:** 1 Hugh Sinclair Unit of Human Nutrition, Department of Food and Nutritional Science, University of Reading, Whiteknights, Pepper Lane, Reading, RG6 6DZ, UK; 2 Institute for Food, Nutrition and Health, University of Reading, Reading, UK

**Keywords:** Dairy biomarkers, Dairy fatty acids, Dairy foods, Heptadecanoic acid, Pentadecanoic acid, Phytanic acid, Trans-palmitoleic acid, Vaccenic acid

## Abstract

In epidemiological studies, dairy food consumption has been associated with minimal effect or decreased risk of some cardiometabolic diseases (CMD). However, current methods of dietary assessment do not provide objective and accurate measures of food intakes. Thus, the identification of valid and reliable biomarkers of dairy product intake is an important challenge to best determine the relationship between dairy consumption and health status. This review investigated potential biomarkers of dairy fat consumption, such as odd-chain, trans- and branched-chain fatty acids (FA), which may improve the assessment of full-fat dairy product consumption. Overall, the current use of serum/plasma FA as biomarkers of dairy fat consumption is mostly based on observational evidence, with a lack of well-controlled, dose–response intervention studies to accurately assess the strength of the relationship. Circulating odd-chain SFA and trans-palmitoleic acid are increasingly studied in relation to CMD risk and seem to be consistently associated with a reduced risk of type 2 diabetes in prospective cohort studies. However, associations with CVD are less clear. Overall, adding less studied FA such as vaccenic and phytanic acids to the current available evidence may provide a more complete assessment of dairy fat intake and minimise potential confounding from endogenous synthesis. Finally, the current evidence base on the direct effect of dairy fatty acids on established biomarkers of CMD risk (e.g. fasting lipid profiles and markers of glycaemic control) mostly derives from cross-sectional, animal and *in vitro* studies and should be strengthened by well-controlled human intervention studies.

CVD are responsible for 26 % of deaths in the UK, and healthcare costs related to CVD represent a £9 billion economic burden annually^(1)^. In parallel, metabolic disorders such as type 2 diabetes (T2D), metabolic syndrome and non-alcoholic fatty liver disease are associated with increased risk of CVD in epidemiological studies^(2–4)^. With 7 million people living with CVD and 4·6 million with T2D in the UK, prevention of these cardiometabolic diseases (CMD) is currently one of the biggest modern challenges for public health^(1,5)^. Whilst some CMD risk factors are not modifiable (e.g. age, sex or genetic makeup), modifying dietary behaviours may constitute an effective strategy for disease risk reduction. In particular, dietary intakes of saturated fat, Na and fruits and vegetables have been extensively studied in relation to CMD prevention and have been targeted by public health recommendations^(6)^. In the UK, the first recommendation on dietary SFA (< 10 % total energy) intake was initially implemented by the National Advisory Committee on Nutritional Education (NACNE) in 1983^(7,8)^ and was reiterated in the 2019 Scientific Advisory Committee in Nutrition report on saturated fat and health which recommended a replacement of SFA with poly-PUFA or MUFA for CVD prevention^(9)^. Furthermore, recent epidemiological evidence suggested the potential different associations of individual SFA on CMD risk, which might reflect their different chain lengths or food matrices in which they are incorporated^(10,11)^.

From a food perspective, dairy products are an interesting food group for the management of SFA entry in the food chain. Dairy products contribute up to 21 % of total SFA intake among British adults, and their consumption (particularly cheese and yogurt) seems to have beneficial associations with some risk markers for CMD such as blood pressure and arterial stiffness^(12,13)^. However, most of the currently available evidence on the consumption of dairy foods and/or their fat content and CMD risk is limited to observational studies which often rely on imprecise methods of dietary assessment. Indeed, the reported intakes from FFQ, diet-diaries or dietary recalls may be subject to underestimation, recall bias and systematic errors from food composition tables, which may impact on the associations between dairy intakes and CMD risk in prospective cohort studies. However, validation studies of FFQ against dietary records or blood and urinary biomarkers, the use of repeated assessments of dietary intakes in longitudinal studies and the integration of novel technological tools have contributed to improving the accuracy of these traditional methods of dietary assessment^(14,15)^. Limited precision when assessing dairy foods consumed within composite dishes (e.g. pizza, bakery items or coffee drinks), which might independently impact CMD risk, is a further shortcoming of these dietary assessment methods that may be overcome by deconstructing dairy-derived ingredients from composite dishes and re-allocating them to dairy food categories, as illustrated in the Prospective Urban Rural Epidemiology (PURE) study^(16)^. Whilst these methodological limitations are not restricted to dairy fat intakes and are commonplace in nutritional science, the integration of data from traditional methods of dietary assessment with more objective biomarkers of intake may improve the accuracy of dietary intake assessments and prediction of disease risk. In particular, reliable circulating biomarker candidates need to be (1) specific to the food source of interest; (2) easy to quantify in the organism; (3) exclusively derived from dietary intake rather than endogenous synthesis and (4) highly correlated to dietary intakes. For example, blood concentrations of vitamin C and carotenoids have been widely used as a proxy for fruit and vegetable intakes and linked with decreased risks of cancer in observational studies^(17)^. Therefore, the identification of biomarkers for the consumption of other food groups, such as fats derived from dairy foods, might provide useful insights into their role in cardiometabolic health by complementing the extensive evidence base already available^(18)^.

The following article will thus aim to review the evidence for a range of FA present in milk and dairy foods as biomarker candidates for dairy fat intake: odd-chain SFA (C15:0 and C17:0), which are traditionally used in nutritional epidemiology, and more novel biomarkers such as ruminant *trans*-FA (*trans*-palmitoleic acid C16:1 *trans*-9 and vaccenic acid C18:1 *trans-11*) and one branched-chain FA (phytanic acid). The physiological role of those FA in the context of cardiometabolic health and possible mechanisms of action will also be discussed.

## Identification of individual fatty acids as biomarkers of dairy fat intake

### Odd-chain fatty acids: pentadecanoic (C15:0) and heptadecanoic (C17:0) acids

#### Presence in dairy milk and other food group

**Fig. 1.**
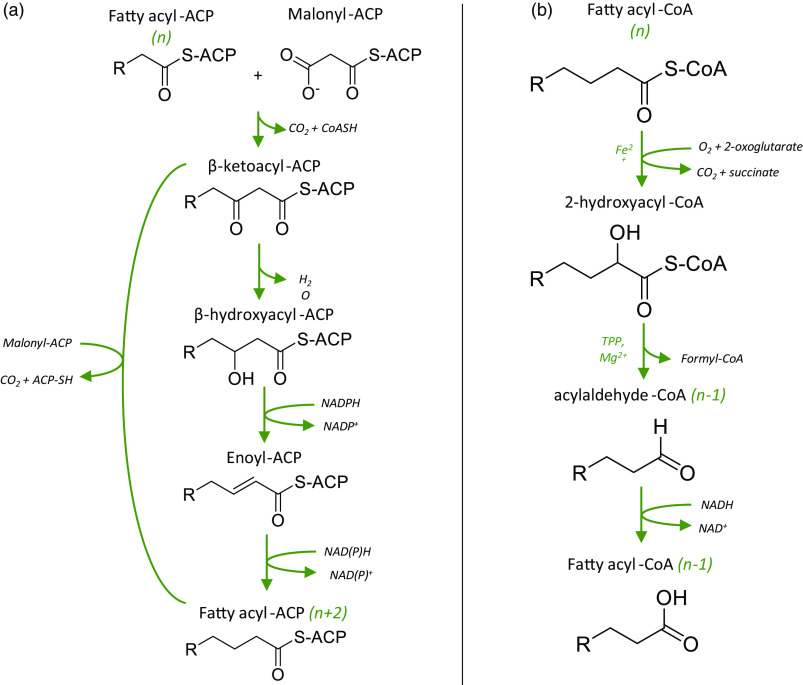
Possible mechanisms for the synthesis of odd-chain SFA. Adapted from Jansen (2006) and Jenkins (2015)^(21,32)^. (a) Metabolic pathway for the synthesis and elongation of even-chain FA (e.g. 16:0, 18:0), starting with the condensation of malonyl-CoA with a fatty acyl-ACP of n carbons. Odd-chain SFA may be produced via the same route, using propionyl-CoA as a precursor instead of malonyl-CoA. (b) Main steps of the α-oxidation of odd-chain fatty acids, involving the decarboxylation of the *α*-carbon to allow further oxidation of the acyl chain. Even-chain SFA may undergo the same decarboxylation reaction, leading to the formation of odd-chain SFA.

In dairy cows and other ruminants, odd-chain FA are produced by bacteria in the rumen and by post-absorptive *de novo* lipogenesis using short-chain FA like propionic acid (C3:0) as a substrate, or by *α*-oxidation which converts C16:0 or C18:0 into C15:0 or C17:0, respectively via the elimination of the *α*-carbon^(19–21)^ (Fig. 1). The produced odd-chain FA are then utilised in the mammary gland for the production of milk fat, although their contribution remains minor in comparison to even-chain FA which are produced via *de novo* lipogenesis at much higher levels^(22)^. Although there are small seasonal variations, SFA account for 67–72 % of total FA in milk and include mostly palmitic (C16:0, 30–33 % total FA), myristic (C14:0, 10–11 % total FA), stearic (C18:0, 9–10 % total FA) acids and short/medium-chain FA (C4:0–C12:0, < 4 % total FA), while odd-chain FA such as pentadecanoic (C15:0) and heptadecanoic (C17:0) represent 1 % and 0·5 % total FA, respectively^(23)^. Thus, one serving of 200 ml of semi-skimmed milk would provide 2 g SFA, of which 20 mg were from C15:0 and 10 mg from C17:0.

Non-dairy dietary sources of C15:0 and C17:0 include ruminant meats and fish, although the proportions of C15:0 from these sources are lower than of C17:0^(24)^. With meat and meat products contributing to as much dietary SFA as dairy foods in British adults (about 21 % of total dietary SFA)^(12)^, the sole use of odd-chain SFA as biomarkers of dairy fat consumption may be questionable especially in the context of diets rich in ruminant meat. However, very limited evidence from the European Prospective Investigation into Cancer and Nutrition-Potsdam^(25)^ and European Prospective Investigation into Cancer and Nutrition-E3N^(26)^ prospective studies has suggested that circulating C15:0 or C17:0 and red meat consumption might be inversely correlated or not correlated, respectively. Moreover, the analysis of the FA content of twenty-seven freshwater fish species revealed that C15:0 and C17:0 represented 0·4 % and 0·6 % total FA, respectively^(27)^, which was also reflected in the analysis of nineteen different European brands of fish oil supplements^(28)^. Thus, further concerns could be raised on the validity of odd-chain SFA as biomarkers of dairy fats in populations consuming high-fish, low-dairy diets. This is not the case for most Western populations, including British adults, among which fish consumption is relatively low (22 g/d on average or 3 % total energy for British adults aged between 19 and 64 years old)^(12)^, but does not account for the intake of fish oil supplements consumed by an estimated 11 % of British adults in 2012^(29)^.

#### Correlations between circulating levels and dietary intakes

Despite their low contribution to total dietary SFA, odd-chain SFA are detectable in low concentrations in human plasma samples but were traditionally used as internal standards in GC analytical methods which masked any possible quantitative assessment^(30,31)^. However, with the growing interest in their utility as biomarkers of dairy fat intake, circulating levels are more routinely measured in a number of plasma FA fractions, such as non-esterified FA or phospholipids^(32)^.

To date, C15:0 and C17:0 have formed the basis for much of the research on biomarkers of dairy fat and specific dairy food group consumption. In particular, findings from two cross-sectional studies indicated a better correlation between circulating C15:0 and high-fat dairy products or total dairy fat compared with lower fat dairy product foods^(33,34)^. In a cross-sectional study of *n* seventy-two participants, C15:0 in plasma phospholipids was positively correlated with fat from both milk and cream (*r* = 0·34) and total dairy fat (*r* = 0·34), but not with butter, ice cream or fat from either^(33)^. More recently, findings from the Food4Me study (*n* 1054 participants) suggested stronger correlations between high-fat dairy food consumptions and C15:0 in dried blood spots compared with C17:0^(34)^. The authors reported positive associations between C15:0 (% change in blood) and consumed daily portions of total dairy (regression coefficient *β* = 1·02, 95 % CI (0·14, 1·91)), high-fat dairy (*β* = 0·32, 95 % CI (0·05, 0·58)), cheese (*β* = 1·77, 95 % CI (0·52, 3·02)) and butter (*β* = 3·34, 95 % CI (1·34, 5·35)). In contrast, C17:0 was associated with intakes of cream (*β* = 9·42, 95 % CI (3·43, 15·4)) and high-fat dairy albeit to a lesser extent than C15:0 (*β* = 0·27, 95 % CI (0·1, 0·45)), but not with total dairy, cheese or butter. Finally, the authors did not report any associations between C15:0 or C17:0 and consumption of low-fat dairy foods, milk or yogurts^(34)^. The discrepancy observed between C15:0 and C17:0 might be partly explained by the biological matrix used for analysis, as suggested by findings from the LifeLines Cohort study (*n* 864 participants) where total dairy product intakes were positively related with both C15:0 and C17:0 in plasma phospholipids, but only C15:0 in plasma TAG)^(35)^. Overall, findings from observational studies tend to support consistent, albeit weak, correlations between dairy product fat intakes and plasma/serum C15:0 (*r* = 0·33, 95 % CI (0·27, 0·39)) and to a lesser extent C17:0 *(r* = 0·19, 95 % CI (0·14, 0·25)), as summarised in a 2019 meta-analysis of eighteen cross-sectional and prospective studies^(36)^. However, these results need to be interpreted in the light of their observational and cross-sectional nature, which prevent causal links between dairy fat productconsumption and circulating C15:0 or C17:0 from being inferred^(37)^.

In line with findings from observational studies, findings from RCT suggest that circulating concentrations of C15:0 are more responsive to dairy fat consumption than concentrations of C17:0^(38–40)^ (Table 1). However, the correlations between total dairy product intake and circulating levels of C15:0 and C17:0 are generally weak, as observed in a 12-month randomised controlled trial (RCT) in seventy-six adolescents which reported modest correlations between total dairy product consumptions (in servings/d) and C15:0 (*r* = 0·27) or C17:0 *(r* = 0·25) measured in erythrocytes^(41)^. Importantly, the designs of the RCT included in this review suggest that most often the hypothesis being tested is whether specific FA, such as C15:0 and C17:0, are biomarkers of dairy food consumption, rather than dairy fat. As suggested by the poor or null correlations observed between C15:0 or C17:0 and intakes of low-fat dairy foods^(33,34)^, this approximation seems misplaced, as the circulating levels of FA would not accurately capture the consumption of dairy foods with a minimal fat content and thus would not reflect the consumption of total dairy accurately.

**Table 1.** Human randomised control trails investigating the correlations between dairy consumption and circulating levels of odd-chain, *trans* and/or branched-chain fatty acids

Reference	Fatty acid(s) of interest	Biological fraction measured	Subject group *n*, sex, mean age, BMI	Study design and duration	Intervention(s)	Comparison(s)	Intervention effect(s)
Vissers *et al*. 2020^(39)^	C15:0, C17:0	Plasma cholesteryl esters	*n* 30 (13M/17F) Age: 22 y BMI: 21·6 kg/m2	Crossover, 1 week	High-dairy diet (14 % dietary protein from dairy foods)	High-meat diet (14 % dietary protein from pork, beef and chicken), High-grain diet (14 % dietary protein from wheat, bran, rice, maize and legumes)	↑ C15:0 *v*. high-meat (*P* = 1·2 × 10^−5^) or high-grain diets (*P* = 1·2 × 10^−2^) ↔ C17:0 *v*. high-meat or high-grain diets
Slim *et al.* 2019^(41)^	C15:0, C17:0, tPA	Plasma total lipids, erythrocytes	Intervention *n* 29 (8M/21F) Age: 16·5 y BMI: 21·7 kg/m2 Control (CTL) 33 (8M/25F) Age: 16·6 y BMI: 21·7 kg/m2	Parallel, 12 months	≥ 4 serv/d of dairy food of choice	≤ 2 serv/d of dairy food of choice (CTL)	Plasma total lipids: ↔ C15:0, C17:0, tPA *v*. CTL Correlations with dairy consumption: NS Erythrocytes: ↑ C15:0 *v*. CTL (*P* < 0·05) ↔ C17:0, tPA *v*. CTL Spearman correlations with dairy consumption: (+) C15:0 (*r* = 0·27), C17:0 (*r* = 0·25) NS tPA
O’Connor *et al*. 2019^(40)^	C15:0, C17:0	Serum total lipids	*n* 26 (19M/7F) Age: 54·9 y BMI: 21·9 kg/m2	Crossover, 18 weeks	≥ 4 serv/d of dairy food of choice	≤ 2 serv/d of dairy food of choice (CTL)	Change from baseline in intervention: ↑ C15:0 (*P* = 0·04) ↔ C17:0 Intervention *v*. CTL: ↑ C17:0 (*P* = 0·045) ↔ C15:0
Abdullah *et al*. 2015^(38)^	C15:0, C17:0, tPA	Plasma total lipids	*n* 124 (40M/84F) Age: 39·3 y BMI: 25·7 kg/m2	Crossover, 4 weeks	3 serv/d of dairy: 375 ml of 1 % fat milk 175 g of 1·5 % fat yogurt 30 g of 34 % fat cheese	Energy-equivalent control (CTL): 290 ml of fruit and vegetable juice 20 g of cashews 39 g of cookie	↑ C15:0, C17:0 *v*. CTL (*P* < 10^−4^) ↔ tPA *v*. CTL
Werner *et al*. 2013^(65)^	C15:0, PhA	Plasma total lipids	Intervention *n* 20 (7M /13F) Age: 61·9 y BMI: 25·4 kg/m2 Control (CTL) 18(8M/10F) Age: 60·7 y BMI: 26·5 kg/m2	Parallel, 12 weeks	39 g/d of fat from high-PhA butter, made from milk of mountain-pasture grazing cows	39 g/d of fat from butter lower in PA, made from milk of cows fed conventional winter fodder (CTL)	↔ PhA *v*. CTL ↑ C15:0 *v*. baseline in both intervention and CTL (*P*-value not reported)
Werner *et al*. 2011^(64)^	PhA	Plasma total lipids	Intervention *n* 9 (3M/6F) Age: 28·3 y BMI: 22·2 kg/m2 Control (CTL) *n* 5 (3M/2F) Age: 31·6 y BMI: 22·4 kg/m2	Parallel, 4 weeks	45 g/d of fat from test butter and cheese with 0·24 wt % PhA	45 g/d of fat from test butter and cheese with 0·13 wt % PhA (CTL)	↔ PhA *v*. CTL ↑ PhA *v*. baseline in both intervention and CTL (*P* < 0·05)

C15:0, pentadecanoic acid; C17:0, heptadecanoic acid; tPA, *trans*-palmitoleic acid; PhA phytanic acid; M, male; F, female; y, year; serv/d, serving per day; g/d, gram per day; % wt, weight percent; CTL, control; ↑, increase; ↔, no change; NS, not significant; (+), positive.

#### Potential limitations for use of C15:0 and C17:0 as biomarkers of dairy fat consumption

In phospholipid FA, levels of C15:0 and C17:0 range from 0·15 to 0·23 % (*n* 4 studies) and 0·33–0·41 % total phospholipid FA (*n* 3 studies), respectively^(32)^. Therefore, although C15:0 is more abundant in milk than C17:0, plasma phospholipid levels seem to indicate an inverse abundance of C15:0 and C17:0 in human plasma, which raises the question of possible alternative pathways for endogenous synthesis and/or oxidation of odd-chain SFA in humans. Proposed mechanisms suggest the human gut microbiota might be involved in the elongation of short-chain FA and/or decarboxylation of even-numbered very-long chain Fas^(32,42)^.

*In vivo* studies in rats suggest the need for a distinction between circulating levels of C15:0, which would be mostly of dietary origin and inversely correlated with total dietary fat, and circulating levels of C17:0, which seem unaffected by total dietary fat and may be endogenously synthesised^(43)^. This hypothesis is in line with recent *in vitro* findings that suggested that the main human elongation of very long-chain fatty acids (ELOVL) enzymes could not only elongate even-chain SFA but could also act on odd-chain FA by catalysing the elongation of C13:0 to C15:0 and C15:0 to C17:0^(44)^. To date, the metabolic mechanisms related to the elongation of odd-chain SFA in humans are not fully elucidated and their contribution to circulating levels have not been precisely quantified.

In addition to potential endogenous synthesis and gut microbiome fermentation, the interactions between odd-chain SFA and other dietary nutrients such as fibre are unclear^(45)^. In particular, in a human RCT of sixteen healthy participants, the consumption of 30 g/d of inulin or 6 g/d of propionate for 7 d increased plasma phospholipid concentrations of C15:0 by 17 % and 13 % and concentrations of C17:0 by 11 % and 13 %^(46)^. The authors suggested that these results may be explained by the activation of odd-chain SFA metabolic pathways in the gut microbiota^(46)^. A potential endogenous synthesis of odd-chain SFA in response to dietary fibre and in the absence of dairy fat consumption would be in line with observations from an Austrian cross-sectional study which reported similar circulating levels of C17:0 in vegan (*n* 37), omnivore (*n* 23), semi-omnivore (*n* 13) and vegetarian (*n* 25) participants^(47)^.

### Trans-fatty acids of ruminant origins: trans-palmitoleic acid (C16:1 trans-9) and vaccenic acid (C18:1 trans-11)

#### Presence in dairy milk and other food groups

**Fig. 2.**
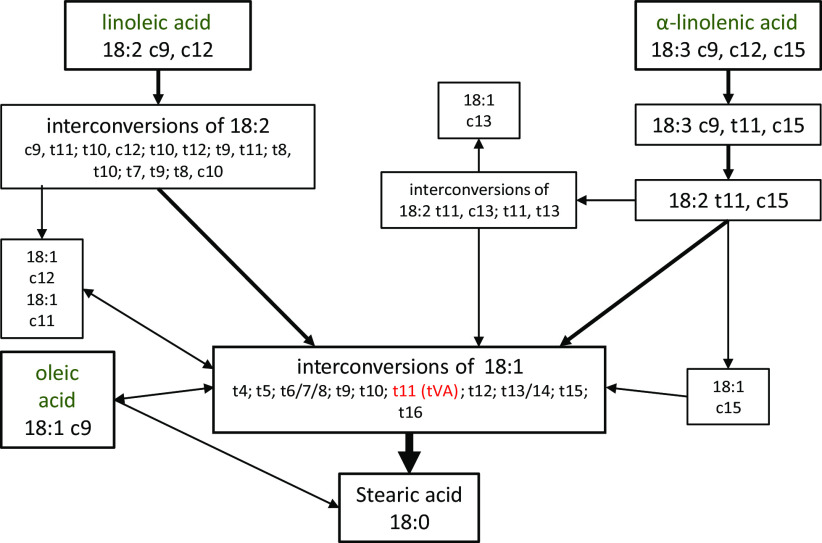
Major biohydrogenation pathways of oleic, linoleic and *α*-linolenic acids into trans-fatty acids in ruminants. Adapted from Enjalbert (2012)^(48)^.

In ruminant animals, dietary FA, such as oleic, linoleic or α-linolenic acids, are utilised by rumen microbiota and undergo a series of biohydrogenation reactions leading to the synthesis of *trans*-FA (Fig. 2)^(48)^. Trans-FA represent 4·5–6·5 % total FA in retail milk, with *trans*-vaccenic acid (tVA, C18:1 *trans*-11) being the major contributor (0·8–1·5 % total FA), followed by *trans*-palmitoleic acid (tPA, C16:1 *trans*-9) representing 0·35–0·45 % total Fas^(23)^. Thus, one serving of 200 ml semi-skimmed milk would provide low doses of tVA and tPA – up to 84 mg total *trans*-FA, including 27 mg tVA and 8·1 mg tPA^(23)^. Similar to odd-chain SFA, tPA and tVA are not entirely specific to dairy foods. However, they belong to the distinct category of ruminant *trans*-FA, as opposed to industrially processed *trans*-FA that derive from the partial hydrogenation of vegetable oils such as elaidic acid (C18:1 *trans*-9).

#### Correlations between circulating levels and dietary intakes

Circulating tPA measured by GC represents < 1 % total phospholipid FA (0·18 ± 0·05 % total FA)^(49)^, although most studies only tend to report concentrations of total *trans*-FA or elaidic acid^(50,51)^. However, one cross-sectional study of 210 healthy Canadian adults reported that tPA and tVA each represented < 0·20 % of total plasma phospholipid FA^(52)^. Circulating tPA levels in this study were weakly correlated with dairy product intakes estimated by an FFQ (*r* = 0·15, *P* = 0·04) but tVA levels were not^(52)^. In contrast, a more recent cross-sectional study from the Netherlands described that circulating tVA in plasma phospholipids had a stronger correlation with dairy fat consumption than odd-chain SFA^(35)^. Overall, very few well-controlled RCT have investigated the associations between dairy fat consumption and circulating ruminant *trans*-FA, and the few that have only reported results on tPA, but not tVA (Table 1).

Several epidemiological studies, which date from the 1990s, observed significant correlations between circulating levels of tPA and non-dairy fat dietary sources such as cakes, cookies and pies rather than dairy fat^(53,54)^. At the time, such products contained hidden sources of dairy fat such as milk powder, butter and chocolate, but also partially hydrogenated oils. Since then, *trans*-fat has mostly been eliminated from the European and American food chains due to the significant link with increased CVD risk^(55)^. Therefore, dietary tPA and tVA now almost entirely originate from dairy foods and ruminant fats, as described by a wide market basket analysis of French foods^(56)^.

#### Potential limitations for use as biomarkers of dairy fat consumption

Findings from a human parallel RCT, which showed a significant increase in tPA circulating levels following dietary intake of tVA (2·9 g/d for 6 weeks), suggested that dietary tVA could be converted into tPA endogenously, with a conversion rate of approximately 17 %^(57)^. These results were in line with *in vitro* studies, suggesting that tVA could be converted into tPA via peroxisomal *β*-oxidation pathways^(58)^. Of note, these findings were challenged in an editorial from Mozaffarian, on the basis that one of the genome-wide association studies his group led failed to reveal significant genetic determinants for the synthesis of tPA, suggesting that this endogenous synthesis would be negligible^(59,60)^. Overall, the limited evidence on potential endogenous synthesis of tPA and tVA raises questions about their suitability as biomarkers of dairy fat consumption.

### Branched-chain fatty acids: phytanic acid

#### Presence in dairy milk and other food groups

Phytanic acid (PhA) is a branched-chain FA with a main chain of sixteen carbons and four methyl groups (3,7,11,15-tetramethylhexadecanoic acid). As with previous FA described in this review, it is found in relatively low quantities in milk (200 ml semi-skimmed milk provides approximately 9·8 mg of PhA), with levels found to be ten times higher in hard cheese (98·9 mg/100 g cheese)^(61)^. However, PhA is also present in relatively high quantities in beef (up to 326 mg/100 g food) and fish (100 mg/100 g halibut or capelin)^(61)^. Furthermore, PhA is the degradation product of phytol, which derives from chlorophyll. Thus, its content in animal-derived products widely varies according to the farming method and animal diet, with a diet rich in grass leading to a higher PhA content in meat and dairy foods. In humans, PhA cannot be endogenously synthesised *de novo* but can be produced in the liver via the conversion of pristanic acid, a branched-chain FA involved in peroxisomal metabolism, although this pathway seems to be minimal^(62)^.

#### Correlations between circulating levels and dietary intakes

A 2010 prospective study from the European Prospective Investigation into Cancer and Nutrition cohort revealed that although PhA levels correlated with dairy product intakes (*r* = 0·49, *P* < 10^−4^), this correlation was influenced by external factors such as age, country of residence and duration of fasting at blood collection^(63)^. To date, very few RCT investigated the correlations between dairy fat intake and circulating PhA levels (Table 1). In 2011, a dietary RCT in humans revealed significant increases in PhA levels after consumption of milk, butter and cheese^(64)^. The authors also reported a significant correlation between the consumption of butter from mountain-pasture grazing cows or cows fed conventional winter fodder and circulating PhA levels^(65)^.

### Summary of findings

Most of the evidence available to identify individual FA as biomarkers of dairy fat intake focused on odd-chain SFA, while findings on tPA, tVA and PhA are much more limited. The lack of well-controlled dose–response studies designed to validate biomarkers of dairy consumption represents an important limitation to their use in epidemiological studies.

## Relevance to cardiometabolic disease risk and potential mechanisms

Circulating FA are often used as proxies for assessing the associations between dairy consumption and cardiometabolic health in observational epidemiological studies, although their physiological role in metabolism and cardiometabolic health is still unclear.

### Odd-chain fattyacids: C15:0 and C17:0

#### Associations with markers of cardiometabolic health

At least six cross-sectional studies observed significant inverse associations between circulating odd-chain SFA, particularly C17:0, and lipid profiles, markers of inflammation or glucose metabolism, although these correlations seem to be observed mostly in overweight and obese participants^(36,66–70)^. Two cross-sectional studies also suggested potential influences of other nutrients such as alcohol or different dietary patterns on the relationship between circulating odd-chain SFA and lipid profiles^(69,71)^. Stronger evidence from well-controlled intervention studies investigating the specific role of odd-chain SFA is still very limited. One recent dairy-feeding RCT reported concomitant increases in odd-chain SFA levels and LDL-cholesterol concentrations after the consumption of three servings of dairy/d for 4 weeks compared with an energy-matching control (290 ml of fruit and vegetable juice, 20 g of cashew nuts and a 39 g of cookie)^(38)^.

#### Associations with CMD risk and mortality

So far, meta-analyses of prospective observational studies on the associations between circulating odd-chain SFA and CVD risk and mortality have yielded contrasting results, suggesting either neutral or inverse associations. Chowdury and colleagues observed a 23 % CHD risk reduction (relative risk (RR) = 0·77; 95 % CI (0·63, 0·93)), *n* 2283 cases/5490 participants) associated with higher circulating levels of C17:0. This association was consistent, albeit not statistically significant, when adding circulating C15:0 (RR = 0·81; 95 % CI (0·62, 1·06)), *n* 2283 cases/5490 participants)^(72)^. However, these findings were not supported by data from a more recent meta-analysis of twelve European and North American prospective studies which included 7680 CVD cases^(73)^. The authors failed to observe a significant association between circulating odd-chain SFA and overall CVD or subtypes of CVD risks, but did reveal a 28 % heart failure risk reduction associated with circulating C15:0 (RR = 0·72; 95 % CI (0·55, 0·95)), *n* 983 cases from two studies)^(73)^. Further prospective human studies that investigated these associations but were not included in the above meta-analyses are summarised in Table 2. Briefly, findings from the prospective Cardiovascular Health Study revealed no changes in CVD risk, but a 33 % reduction in CVD mortality risk (hazard ratio HR = 0·77; 95 % CI (0·61, 0·98)), *n* 833 CVD deaths/1595 non-CVD deaths) associated with the highest quintile of circulating C17:0 compared with the lowest quintile (median concentration 0·48 % *v*. 0·31 % total plasma FA, respectively) after a 22-year follow-up. However, circulating C15:0 was not associated with CVD risk or mortality in this cohort^(74)^. In contrast, three prospective studies failed to observe associations between circulating C17:0 and CVD risk but did report inverse associations between C15:0 and risks of myocardial infarction and stroke^(70,74,75)^.

**Table 2.** Prospective human studies investigating the associations between circulating levels of odd-chain or *trans*-fatty acids and incident CVD, CVD mortality or incident type 2 diabetes (T2D)

Reference	Fatty acid(s) of interest	biological fraction measured	Reported study and overall participants characteristics (e.g. *n*, sex, mean age, mean BMI)	Study design and mean follow-up	Outcomes	No. cases and/or no. of deaths	Confounders included	Summary of observed associations by fatty acid of interest*
Incident CVD and CVD mortality								
De Oliveira Otto *et al*. (2018)^(74)^	15:0, 17:0, tPA (5^th^ *v*. 1^st^ quintile)	Plasma phospholipids	Cardiovascular Health Study (USA) *n* 2907 (36 % M, 64 % F) Age: 74·8 y	Prospective cohort study, 22 y	Incident CVD, CHD and stroke total and CVD mortality	1301 CVD 876 CHD 529 strokes	Age, sex, race, education, enrolment site at baseline, smoking status, alcohol, PA, BMI, drug-related hypertension, self-reported general health, circulating total *trans*-FA, consumption of dairy, dietary fibre, fruits, vegetables and red meat	C15:0, C:17:0 tPA ↔ CVD risk ↔ CHD risk ↔ Stroke risk
						2428 deaths 614 CVD deaths		C15:0, tPA ↔ Total mortality ↔ CVD mortality C17:0 ↔ Total mortality ↓ CVD mortality (HR = 0·77 (95 % CI 0·61, 0·98))
Laursen *et al*. (2018)^(75)^	15:0, 17:0, tVA (95^th^ percentile *v*. 5^th^percentile)	Adipose tissue	Danish Diet, Cancer and Health (Denmark) Cases (incident stroke) *n* 2108 (60·5 % M, 39·5 % F) Age: 60·5 y BMI: 26·2 kg/m2 Non-cases *n* 3186 (54 % M, 46 % F) Age: 56·3 y, BMI: 25·8 kg/m2	Case-cohort study, 12·8 y	incident stroke and stroke subtypes	2108 total strokes 1745 ischaemic strokes 249 intracerebral haemorrhages (IH) 102 subarachnoid haemorrhages (SH)	Sex, date of inclusion, education, BMI, waist circumference, PA, smoking status, alcohol intake, baseline hypertension, hypercholesterolemia, diabetes and myocardial infarction	C15:0 ↓ Total stroke (HR = 0·59 (95 % CI 0·47, 0·74)) ↓ Ischaemic stroke (HR = 0·55 (95 % CI 0·43, 0·71)) ↔ IH, SH C17:0 ↔ Total stroke, IH, SH ↓ Ischaemic stroke (HR = 0·74 (95 % CI 0·58, 0·94)) tVA ↓Total stroke (HR = 0·34 (95 % CI 0·27, 0·44)) ↓ Ischaemic stroke (HR = 0·30; 95 % CI (0·24, 0·39)) ↓ IH (HR = 0·45; 95 % CI (0·26, 0·78)) ↔ SH
Kleber *et al*. (2016)^(78)^	tPA (3^rd^ *v*. 1^st^ tertile)	Erythrocytes	Ludwigshafen Risk and Cardiovascular Health Study (Germany) *n* 3259 (69·7 % M, 30·3 % F) Age: 62·7 y BMI: 27·5 kg/m2 Patients hospitalised for coronary angiography	Prospective cohort study, 10 y	All-cause and CVD mortality	975 deaths 614 CVD deaths 254 sudden cardiac deaths	Age, sex, BMI, LDL-C, HDL-C, log-transformed TAG, log-transformed fibrinogen, smoking status, hypertension, diabetes, lipid-lowering therapy, glomerular filtration rate, HbA1c, anti-hypertensive medication, alcohol intake	tPA ↔ All-cause mortality, CVD mortality ↓ Sudden cardiac death (HR = 0·65; 95 % CI (0·47, 0·90))
Warensjö *et al*. (2003) ^(70)^	C15:0, C170, C15:0 + C17:0 (continuous)	Serum cholesteryl esters, Serum phospholipids	Northern Sweden Health and Disease Study *n* 1000 (61·5 % M, 38·5 % F) Age: 49–64y BMI: 23·2–29·4 kg/m2	Nested prospective case–control study, 3·1–3·9 y	Incident myocardial infarction	444 cases 556 controls	PA, BMI, smoking status, intakes of fruits and vegetables, education, ApoB/ApoA-I ratio, systolic blood pressure, BMI, prevalence of diabetes	C15:0, C170, C15:0 + C17:0 ↔ Myocardial infarction risk
Incident T2D								
Liu *et al*. (2018)^(77)^	C15:0, C17:0, C15:0 + C17:0 (continuous)	Calculated dietary intakes from FFQ	European Prospective Investigation into Cancer and Nutrition-Netherlands *n* 37 421 (25·6 % M, 74·4 % F) Age: 49 y BMI: 25·3–26·0 kg/m2 across quartiles of dietary SFA	Prospective cohort study, 10·1 y	Incident T2D	893 T2D cases	Sex, age, sum of other SFA, education, smoking status, PA, BMI, waist circumference, energy-adjusted dietary intakes of: alcohol, animal protein, vegetable protein, *trans*-FA, vitamin E, fibre, cholesterol	C15:0 ↔ T2D C17:0 ↓ T2D (HR = 0·84; 95 % CI (0·73, 0·97)) C15:0 + C17:0 ↓ T2D (HR = 0·88; 95 % CI (0·79, 0·99))
Liu *et al*. (2018)^(79)^	tPA, tVA (5^th^ *v*. 1^st^ quintile)	Plasma total lipids	National Health and Nutrition Examination Survey (USA) *n* 3801 (48 % M, 52 % F) Age: 50·1 y (M), 50·0 y (F)	Prospective cohort study, 11 y	Incident T2D	505 T2D cases	Age, gender, race/ethnicity, education, family income, smoking status, PA, alcohol intake, family history of diabetes, total energy intake, Healthy Eating Index-2010, BMI	Tpa ↔ T2D (OR = 1·37; 95 % CI (0·90, 2·06)) tVA ↔ T2D (OR = 1·37; 95 % CI (0·95, 1·99))

C15:0, pentadecanoic acid; C17:0, heptadecanoic acid; tPA, *trans*-palmitoleic acid; M, male; F, female; y, year; T2D, type 2 diabetes; IH, intracerebral haemorrhage; SH, subarachnoid haemorrhage; Apo, apolipoprotein; FA, fatty acids; PA, physical activity; ↑, direct association; ↓, inverse association; ↔, no association.

* HR and OR presented as estimate (95 % confidence interval).

Many prospective cohorts have assessed the relationships between circulating odd-chain SFA and risk of T2D, as summarised in a large meta-analysis of sixteen prospective cohorts and which included > 60 000 participants^(76)^. In this pooled analysis, the authors observed inverse associations between T2D risk and both circulating C15:0 (HR = 0·80; 95 % CI (0·73, 0·87)) and C17:0 (HR = 0·65; 95 % CI (0·59, 0·72)). These findings were in line with one prospective cohort published since then that was based on dietary intakes of odd-chain SFA rather than circulating levels, which observed a 12 % T2D risk reduction associated with every additional 0·11 % total energy from odd-chain SFA^(77)^.

#### Potential physiological role(s) in cardiometabolic health

As previously described, odd-chain SFA metabolism in humans may involve gut microbiota and interactions with other dietary components such as fibre^(46)^. In addition, it has been suggested that they may contribute to mitochondrial metabolism and to the synthesis of very-long-chain SFA^(42)^. Thus, the action of odd-chain SFA might have an indirect effect on CMD risk.

### Trans-fatty acids: trans-palmitoleic and vaccenic acids

#### Associations with markers of cardiometabolic health

Cross-sectional studies have observed inverse correlations between circulating levels of tPA and systolic blood pressure^(52)^, fasting glucose^(68)^ and plasma TAG^(35)^. These studies also suggested inverse correlations between tVA and BMI (in men only)^(52)^, fasting glucose and C-reactive protein concentrations^(35)^. In addition, four cohort studies determined cross-sectional associations between baseline levels of tPA and CMD risk biomarkers. Higher circulating tPA levels were associated in three of these studies with elevated LDL-cholesterol and HDL-cholesterol concentrations, but lower TAG and biomarkers of T2D risk (i.e. fasting glucose, fasting insulin and insulin resistance index)^(49,53,78)^. In contrast, a more recent prospective cohort study observed direct cross-sectional relationships between higher circulating tPA and tVA and markers of glycaemic control (i.e. fasting glucose and insulin, HOMA-IR and HbA1c)^(79)^. Overall, most of these observations suggest inverse correlations between tPA and TAG together with direct correlations with LDL-cholesterol and HDL-cholesterol. To date, only one crossover RCT (*n* 106) reported that replacing dietary stearic acid with ruminant tVA or industrial *trans*-FA led to a similar increase in atherogenic lipids such as LDL-cholesterol, apolipoprotein B and lipoprotein (a)^(80)^.

#### Associations with CMD risk and mortality

A 2018 cross-sectional study (*n* 3504) reported an inverse correlation between CVD risk and circulating tPA, but not tVA^(81)^. Prior to this, stronger observational evidence from a meta-analysis of prospective studies observed a trend for inverse associations between tPA levels and overall CVD risk (RR = 0·82; 95 % CI (0·67, 1·02)) and significant inverse associations with T2D risk (RR = 0·58; 95 % CI (0·46, 0·74), *P* < 0·001)^(82)^. The latter observation was in line with results from a more recent larger meta-analysis of sixteen prospective cohort studies, which found that circulating tPA was associated with an 18 % reduced risk of T2D (HR = 0·82; 95 % CI (0·70, 0·96))^(76)^.

Since then, two prospective cohort studies observed inverse associations between circulating tVA and risk of strokes^(75)^, or between tPA and sudden cardiac mortality^(78)^, but other studies failed to observe associations between tPA and CVD risk^(74)^ or between tVA and tPA and risk of T2D^(79)^ (Table 2).

#### Potential physiological role(s) in cardiometabolic health

A number of mechanisms have been proposed from *in vitro* and animal studies to explain a potential impact of tPA and tVA on health outcomes. *In vitro* treatment of human endothelial cells with tPA and tVA (50 μmol/l for 24 h) was reported to significantly decrease TNF*α*-induced prostaglandin excretion and inflammatory gene expression, thus potentially limiting low-grade inflammation in blood vessels^(83)^. Second, tPA may have similar functions as its *cis*-isomer palmitoleic acid, which has been shown to protect animals from diabetes via the improvement of insulin sensitivity and reduction of hepatic *de novo* lipogenesis^(53,84)^. Similar improvements in markers of glycaemic control have been observed in obese and diabetic rats supplemented with tVA^(85,86)^.

### Phytanic acid

To our knowledge, there is no epidemiological data on the associations between dietary or circulating PhA and CMD risk markers or clinical events. A small double-blind, parallel trial (*n* 5 in control group, *n* 9 in intervention group) failed to reveal any significant changes in blood lipids, C-reactive protein, insulin or glucose levels after chronic consumption of PhA-enriched milk, butter and cheese for 4 weeks^(64)^.

Hypotheses regarding PhA in human metabolism and health have been proposed in a number of narrative reviews and mostly derive from *in vitro* and animal studies^(61,87)^. For example, diets enriched in phytol, a precursor for PhA, led to significant weight loss and reductions in serum total lipids, TAG and cholesterol esters in male mice^(88)^. Similarly, obese mice fed with high-fat diets enriched in phytol showed reduced plasma TAG, hepatic TAG accumulation and obesity-induced fatty liver via the activation of peroxisome proliferator-activated receptor-*α* (PPAR*α*)^(89)^. Recent *in vitro* findings have suggested that the activation of PPAR*α* may also reduce deleterious adiposity by promoting the differentiation of preadipocyte cells into beige adipocytes^(90)^.

### Summary of findings

Overall, the evidence linking circulating odd-chain SFA, tPA and tVA with cardiometabolic health status or CMD events is mostly based on observational studies that reported contrasting results, while only one RCT investigated the effect of PhA on markers of CMD risk. In addition, the physiological role of these FA and their potential importance in CMD aetiology have not been previously investigated in humans, warranting further research on this topic.

## Discussion

Dairy food consumption has often been associated with minimal or decreased CMD risk in epidemiological studies, but current methods of dietary assessment do not provide objective and reliable measures of intake. Thus, the identification of valid and reliable biomarkers of dairy fat intake is an important challenge to best determine the relationship between dairy fat consumption and cardiometabolic health. This review investigated potential biomarker candidates of dairy fat, such as odd-chain, *trans*- and branched-chain FA.

According to the available literature, circulating levels of odd-chain SFA seem to be modestly associated with consumptions of high-fat dairy foods in most populations. Limitations include potential endogenous synthesis and contributions of other dietary sources of these FA, which crucially need to be further assessed and ideally quantified. Moreover, the reliability of odd-chain SFA as biomarkers of dietary dairy fat in the context of different dietary patterns (e.g. low-dairy, high-fish, high-meat, high-fibre or vegan diets) is still unclear. While associations between odd-chain SFA and CVD risk are still unknown, potential associations are more likely to be driven by circulating C17:0 than C15:0. Meanwhile, the evidence on T2D appears to be more in agreement and suggests an inverse association between circulating odd-chain SFA and risk of T2D. In general, findings from circulating odd-chain SFA, which cannot discriminate specific types of dairy foods consumed, are broadly supportive of those from observational studies based on traditional dietary assessment methods^(91)^. However, these studies highlight that differential associations may exist between specific dairy foods consumed in the diet and CVD risk and may also be reflective of their overall fat content^(94,95)^. Importantly, although attempts have been made to quantify dairy fat consumption using traditional dietary assessment methods such as FFQ in prospective cohort studies^(92,93)^, future systematic literature reviews and pooled meta-analyses are warranted to assess the consistency between biomarker and dietary-based approaches to investigate the impact of dairy fat on CMD risk.

Interestingly, prospective cohort studies have reported significant correlations between circulating levels of C15:0, C17:0 and tPA (ranging from *r* = 0·3 to 0·8), suggesting the utility of tPA and potentially other ruminant *trans*-FA as novel biomarkers of dairy fat intake^(76)^. However, with mostly outdated results from observational studies on ruminant *trans*-FA, there is a striking need for new investigations on the validity of tPA and tVA as biomarkers of dairy fat intakes, especially since circulating levels of tPA are gaining interest in relation to cardiometabolic health^(59)^. Conversion pathways between the two FA may also lead to confounding if used as biomarkers of dairy fat consumption separately, although the topic seems to be contentious among experts in the field. In the light of the current evidence, no conclusions can be drawn on the relevance of these FA to be used as proxies for dairy fat consumption until more well-controlled dose–response research in humans is conducted. Moreover, the observational evidence on circulating ruminant tPA and tVA in relation to cardiometabolic health does not provide a clear picture, with scarcer evidence on tVA than on tPA. Updated meta-analyses are warranted to confirm the inverse associations observed between circulating tPA levels and T2D risk.

Similar to *trans*-FA, the correlation between circulating concentrations of PhA and consumption of dairy products or dairy fat is still mostly unknown and has not been compared with other potential dietary sources such as fish and meat. While, to our current knowledge, PhA might not represent the most accurate biomarker of dairy fat, its utility might lay in the reflection of different cow feeding regimes (pasture grazing *v*. conventional fodder) and ultimately be used as a more refined biomarker of dairy food quality in combination with more robust biomarkers of intake. Besides, the current lack of data on the direct role of PhA in human cardiometabolic health highlights an important need for research in this direction.

Overall, it is still unclear whether these FA exert a direct effect on cardiometabolic health, or if they only reflect other beneficial components of dairy foods such as bioactive peptides^(96)^. It may also be difficult to disentangle the potential physiological roles of FA from those of the rest of the dairy food matrix, which is highly variable among dairy food groups such as cheese and butter^(97,98)^. In addition, it has been proposed that fermented dairy foods such as yogurts may help prevent obesity via beneficial effects on the gut microbiota, the intestinal barrier function and the hormonal regulation of appetite^(99)^ and may be associated with lower risks of cerebrovascular diseases^(100)^.

### Conclusion

The evidence reviewed here indicates that FA are often considered as biomarkers of dairy consumption by researchers, rather than biomarkers of dairy fat intakes. This may contribute to an important confounding in the interpretation of the results, since FA, if validated as reliable biomarkers, may only accurately estimate the consumption of full-fat dairy products. With current public health guidelines often promoting the consumption of low-fat dairy, and prospective cohorts relying on FFQ that may not always distinguish dairy foods according to their fat content, it is crucial to describe FA biomarkers precisely in research papers to avoid confusion within and outside the scientific community. Importantly, the utility of FA as biomarkers of dairy fat intake needs to be interpreted in the context of overall dietary patterns and potential interactions with other food groups or nutrients.

Overall, well-controlled intervention studies would help strengthen the evidence base on the impact of dairy FA on traditional biomarkers of CMD risk (such as fasting lipid profiles and markers of glycaemic control), which are easily measured and responsive to dietary changes in a matter of a few weeks. Furthermore, the use of combinations rather than individual FA, and insights from -omics methods such as lipidomic, proteomic and metabolomics analyses, may be helpful to identify and validate new biomarkers of dairy fat and total dairy intakes^(101)^. Finally, research in precision nutrition and nutrigenetics have so far showed mixed associations between dairy foods, lactase persistence and cardiometabolic health^(102)^, and further studies which take into account phenotypical traits (e.g. sex, age, adiposity or physical activity levels) may improve the overall understanding of the relationship between dairy and cardiometabolic health. In the future, validated approaches to identify new functional biomarkers of dairy fat consumption may improve our understanding of the relationship between dairy fat intake and cardiometabolic health and contribute to a better assessment of adherence to public health dietary guidelines.
