# Can individual fatty acids be used as functional biomarkers of dairy fat consumption in relation to cardiometabolic health? A narrative review—CORRIGENDUM

**DOI:** 10.1017/S000711452200109X

**Published:** 2022-12-28

**Authors:** Laury Sellem, Kim G. Jackson, Laura Paper, Ian D. Givens, Julie A. Lovegrove

**Details of correction:** reformatted Table 2 supplied

**Existing text:**

See Table 2

**Table 2.** Prospective human studies investigating the associations between circulating levels of odd-chain or *trans*-fatty acids and incident CVD, CVD mortality or incident type 2 diabetes (T2D)

Reference	Fatty acid(s) of interest	biological fraction measured	Reported study and overall participants characteristics (e.g. *n*, sex, mean age, mean BMI)	Study design and mean follow-up	Outcomes	No. cases and/or no. of deaths	Confounders included	Summary of observed associations by fatty acid of interest*
Incident CVD and CVD mortality								
De Oliveira Otto *et al*. (2018)^(74)^	15:0, 17:0, tPA (5^th^ *v*. 1^st^ quintile)	Plasma phospholipids	Cardiovascular Health Study (USA) *n* 2907 (36 % M, 64 % F) Age: 74·8 y	Prospective cohort study, 22 y	Incident CVD, CHD and stroke total and CVD mortality	1301 CVD 876 CHD 529 strokes	Age, sex, race, education, enrolment site at baseline, smoking status, alcohol, PA, BMI, drug-related hypertension, self-reported general health, circulating total *trans*-FA, consumption of dairy, dietary fibre, fruits, vegetables and red meat	C15:0, C:17:0 tPA ↔ CVD risk ↔ CHD risk ↔ Stroke risk
						2428 deaths 614 CVD deaths		C15:0, tPA ↔ Total mortality ↔ CVD mortality C17:0 ↔ Total mortality ↓ CVD mortality (HR = 0·77 (95 % CI 0·61, 0·98))
Laursen *et al*. (2018)^(75)^	15:0, 17:0, tVA (95^th^ percentile *v*. 5^th^percentile)	Adipose tissue	Danish Diet, Cancer and Health (Denmark) Cases (incident stroke) *n* 2108 (60·5 % M, 39·5 % F) Age: 60·5 y BMI: 26·2 kg/m2 Non-cases *n* 3186 (54 % M, 46 % F) Age: 56·3 y, BMI: 25·8 kg/m2	Case-cohort study, 12·8 y	incident stroke and stroke subtypes	2108 total strokes 1745 ischaemic strokes 249 intracerebral haemorrhages (IH) 102 subarachnoid haemorrhages (SH)	Sex, date of inclusion, education, BMI, waist circumference, PA, smoking status, alcohol intake, baseline hypertension, hypercholesterolemia, diabetes and myocardial infarction	C15:0 ↓ Total stroke (HR = 0·59 (95 % CI 0·47, 0·74)) ↓ Ischaemic stroke (HR = 0·55 (95 % CI 0·43, 0·71)) ↔ IH, SH C17:0 ↔ Total stroke, IH, SH ↓ Ischaemic stroke (HR = 0·74 (95 % CI 0·58, 0·94)) tVA ↓Total stroke (HR = 0·34 (95 % CI 0·27, 0·44)) ↓ Ischaemic stroke (HR = 0·30; 95 % CI (0·24, 0·39)) ↓ IH (HR = 0·45; 95 % CI (0·26, 0·78)) ↔ SH
Kleber *et al*. (2016)^(78)^	tPA (3^rd^ *v*. 1^st^ tertile)	Erythrocytes	Ludwigshafen Risk and Cardiovascular Health Study (Germany) *n* 3259 (69·7 % M, 30·3 % F) Age: 62·7 y BMI: 27·5 kg/m2 Patients hospitalised for coronary angiography	Prospective cohort study, 10 y	All-cause and CVD mortality	975 deaths 614 CVD deaths 254 sudden cardiac deaths	Age, sex, BMI, LDL-C, HDL-C, log-transformed TAG, log-transformed fibrinogen, smoking status, hypertension, diabetes, lipid-lowering therapy, glomerular filtration rate, HbA1c, anti-hypertensive medication, alcohol intake	tPA ↔ All-cause mortality, CVD mortality ↓ Sudden cardiac death (HR = 0·65; 95 % CI (0·47, 0·90))
Warensjö *et al*. (2003)^(70)^	C15:0, C170		C15:0 + C17:0 (continuous)	Serum cholesteryl esters, Serum phospholipids	Northern Sweden Health and Disease Study *n* 1000 (61·5 % M, 38·5 % F) Age: 49–64y BMI: 23·2–29·4 kg/m2	Nested prospective case–control study, 3·1–3·9 y	Incident myocardial infarction	444 cases 556 controls
PA, BMI, smoking status, intakes of fruits and vegetables, education, ApoB/ApoA-I ratio, systolic blood pressure, BMI, prevalence of diabetes	C15:0, C170		C15:0 + C17:0 ↔ Myocardial infarction risk					
Incident T2D								
Liu *et al*. (2018)^(77)^	C15:0, C17:0		C15:0 + C17:0 (continuous)	Calculated dietary intakes from FFQ	European Prospective Investigation into Cancer and Nutrition-Netherlands *n* 37 421 (25·6 % M, 74·4 % F) Age: 49 y BMI: 25·3–26·0 kg/m2 across quartiles of dietary SFA	Prospective cohort study, 10·1 y	Incident T2D	893 T2D cases
Sex, age, sum of other SFA, education, smoking status, PA, BMI, waist circumference, energy-adjusted dietary intakes of: alcohol, animal protein, vegetable protein, *trans*-FA, vitamin E, fibre, cholesterol	C15:0 ↔ T2D C17:0 ↓ T2D (HR = 0·84; 95 % CI (0·73, 0·97)) C15:0 + C17:0 ↓ T2D (HR = 0·88; 95 % CI (0·79, 0·99))							
Liu *et al*. (2018)^(79)^	tPA, tVA (5^th^ *v*. 1^st^ quintile)	Plasma total lipids	National Health and Nutrition Examination Survey (USA) *n* 3801 (48 % M, 52 % F) Age: 50·1 y (M), 50·0 y (F)	Prospective cohort study, 11 y	Incident T2D	505 T2D cases	Age, gender, race/ethnicity, education, family income, smoking status, PA, alcohol intake, family history of diabetes, total energy intake, Healthy Eating Index-2010, BMI	Tpa ↔ T2D (OR = 1·37; 95 % CI (0·90, 2·06)) tVA ↔ T2D (OR = 1·37; 95 % CI (0·95, 1·99))

C15:0, pentadecanoic acid; C17:0, heptadecanoic acid; tPA, *trans*-palmitoleic acid; M, male; F, female; y, year; T2D, type 2 diabetes; IH, intracerebral haemorrhage; SH, subarachnoid haemorrhage; Apo, apolipoprotein; FA, fatty acids; PA, physical activity; ↑, direct association; ↓, inverse association; ↔, no association.

* HR and OR presented as estimate (95 % confidence interval).

**Corrected text should read:**

See updated and reformatted Table 2

**Table 2.** Prospective human studies investigating the associations between circulating levels of odd-chain or *trans*-fatty acids and incident CVD, CVD mortality or incident type 2 diabetes (T2D)

Reference	Fatty acid(s) of interest	biological fraction measured	Reported study and overall participants characteristics (e.g. *n*, sex, mean age, mean BMI)	Study design and mean follow-up	Outcomes	No. cases and/or no. of deaths	Confounders included	Summary of observed associations by fatty acid of interest*
Incident CVD and CVD mortality								
De Oliveira Otto *et al*. (2018)^(74)^	15:0, 17:0, tPA (5^th^ *v*. 1^st^ quintile)	Plasma phospholipids	Cardiovascular Health Study (USA) *n* 2907 (36 % M, 64 % F) Age: 74·8 y	Prospective cohort study, 22 y	Incident CVD, CHD and stroke total and CVD mortality	1301 CVD 876 CHD 529 strokes	Age, sex, race, education, enrolment site at baseline, smoking status, alcohol, PA, BMI, drug-related hypertension, self-reported general health, circulating total *trans*-FA, consumption of dairy, dietary fibre, fruits, vegetables and red meat	C15:0, C:17:0 tPA ↔ CVD risk ↔ CHD risk ↔ Stroke risk
						2428 deaths 614 CVD deaths		C15:0, tPA ↔ Total mortality ↔ CVD mortality C17:0 ↔ Total mortality ↓ CVD mortality (HR = 0·77 (95 % CI 0·61, 0·98))
Laursen *et al*. (2018)^(75)^	15:0, 17:0, tVA (95^th^ percentile *v*. 5^th^percentile)	Adipose tissue	Danish Diet, Cancer and Health (Denmark) Cases (incident stroke) *n* 2108 (60·5 % M, 39·5 % F) Age: 60·5 y BMI: 26·2 kg/m2 Non-cases *n* 3186 (54 % M, 46 % F) Age: 56·3 y, BMI: 25·8 kg/m2	Case-cohort study, 12·8 y	incident stroke and stroke subtypes	2108 total strokes 1745 ischaemic strokes 249 intracerebral haemorrhages (IH) 102 subarachnoid haemorrhages (SH)	Sex, date of inclusion, education, BMI, waist circumference, PA, smoking status, alcohol intake, baseline hypertension, hypercholesterolemia, diabetes and myocardial infarction	C15:0 ↓ Total stroke (HR = 0·59 (95 % CI 0·47, 0·74)) ↓ Ischaemic stroke (HR = 0·55 (95 % CI 0·43, 0·71)) ↔ IH, SH C17:0 ↔ Total stroke, IH, SH ↓ Ischaemic stroke (HR = 0·74 (95 % CI 0·58, 0·94)) tVA ↓Total stroke (HR = 0·34 (95 % CI 0·27, 0·44)) ↓ Ischaemic stroke (HR = 0·30; 95 % CI (0·24, 0·39)) ↓ IH (HR = 0·45; 95 % CI (0·26, 0·78)) ↔ SH
Kleber *et al*. (2016)^(78)^	tPA (3^rd^ *v*. 1^st^ tertile)	Erythrocytes	Ludwigshafen Risk and Cardiovascular Health Study (Germany) *n* 3259 (69·7 % M, 30·3 % F) Age: 62·7 y BMI: 27·5 kg/m2 Patients hospitalised for coronary angiography	Prospective cohort study, 10 y	All-cause and CVD mortality	975 deaths 614 CVD deaths 254 sudden cardiac deaths	Age, sex, BMI, LDL-C, HDL-C, log-transformed TAG, log-transformed fibrinogen, smoking status, hypertension, diabetes, lipid-lowering therapy, glomerular filtration rate, HbA1c, anti-hypertensive medication, alcohol intake	tPA ↔ All-cause mortality, CVD mortality ↓ Sudden cardiac death (HR = 0·65; 95 % CI (0·47, 0·90))
Warensjö *et al*. (2003)^(70)^	C15:0, C170, C15:0 + C17:0 (continuous)	Serum cholesteryl esters, Serum phospholipids	Northern Sweden Health and Disease Study *n* 1000 (61·5 % M, 38·5 % F) Age: 49–64y BMI: 23·2–29·4 kg/m2	Nested prospective case–control study, 3·1–3·9 y	Incident myocardial infarction	444 cases 556 controls	PA, BMI, smoking status, intakes of fruits and vegetables, education, ApoB/ApoA-I ratio, systolic blood pressure, BMI, prevalence of diabetes	C15:0, C170, C15:0 + C17:0 ↔ Myocardial infarction risk
Incident T2D								
Liu *et al*. (2018)^(77)^	C15:0, C17:0, C15:0 + C17:0 (continuous)	Calculated dietary intakes from FFQ	European Prospective Investigation into Cancer and Nutrition-Netherlands *n* 37 421 (25·6 % M, 74·4 % F) Age: 49 y BMI: 25·3–26·0 kg/m2 across quartiles of dietary SFA	Prospective cohort study, 10·1 y	Incident T2D	893 T2D cases	Sex, age, sum of other SFA, education, smoking status, PA, BMI, waist circumference, energy-adjusted dietary intakes of: alcohol, animal protein, vegetable protein, *trans*-FA, vitamin E, fibre, cholesterol	C15:0 ↔ T2D C17:0 ↓ T2D (HR = 0·84; 95 % CI (0·73, 0·97)) C15:0 + C17:0 ↓ T2D (HR = 0·88; 95 % CI (0·79, 0·99))
Liu *et al*. (2018)^(79)^	tPA, tVA (5^th^ *v*. 1^st^ quintile)	Plasma total lipids	National Health and Nutrition Examination Survey (USA) *n* 3801 (48 % M, 52 % F) Age: 50·1 y (M), 50·0 y (F)	Prospective cohort study, 11 y	Incident T2D	505 T2D cases	Age, gender, race/ethnicity, education, family income, smoking status, PA, alcohol intake, family history of diabetes, total energy intake, Healthy Eating Index-2010, BMI	Tpa ↔ T2D (OR = 1·37; 95 % CI (0·90, 2·06)) tVA ↔ T2D (OR = 1·37; 95 % CI (0·95, 1·99))

C15:0, pentadecanoic acid; C17:0, heptadecanoic acid; tPA, *trans*-palmitoleic acid; M, male; F, female; y, year; T2D, type 2 diabetes; IH, intracerebral haemorrhage; SH, subarachnoid haemorrhage; Apo, apolipoprotein; FA, fatty acids; PA, physical activity; ↑, direct association; ↓, inverse association; ↔, no association.

* HR and OR presented as estimate (95 % confidence interval).

